# ST-Elevation Myocardial Infarction in a 23-Year-Old Female: The Mystery of Thrombus Formation

**DOI:** 10.7759/cureus.15302

**Published:** 2021-05-28

**Authors:** Billal H Sikandar, Scott Butler, Anusha Battula, Rajendra Shetty

**Affiliations:** 1 Internal Medicine, University of Maryland Capital Region Medical Center, Largo, USA; 2 Cardiology, University of Maryland Medical Center, Baltimore, USA; 3 Interventional Cardiology, University of Maryland Capital Region Medical Center, Largo, USA

**Keywords:** st-segment elevation myocardial infarction (stemi), spontaneous coronary artery thrombosis, coronary artery thrombus, covid-19 outbreak, phentermine, coronary artery vasospasm, covid and heart, hyper coagulable state, asymptomatic covid-19, sar-cov 2 infection

## Abstract

Acute ST-elevation myocardial infarction (STEMI) is rarely seen in young adults, however, when encountered, the underlying cause is either a genetic condition leading to early-onset coronary artery disease (CAD), an acquired pro-thrombotic condition, or an idiopathic condition like spontaneous coronary artery dissection (SCAD). Our case describes a healthy 23-year-old female who presented with sudden onset severe angina and was found to have a laminated thrombus in the left anterior descending coronary artery (LAD), with no evidence of intraluminal dissection or plaque rupture. Although the underlying etiology of thrombus formation remains unknown, coronavirus disease 2019 (COVID-19) related thrombotic event is the prime suspect. In addition, another culprit that cannot be excluded is phentermine-induced coronary vasospasm, a commercially available medication for weight loss. This report addresses current literature on acute coronary syndromes in young adults and reviews the potential etiologies for coronary artery thrombosis, which led to a near-fatal acute coronary syndrome in our patient.

## Introduction

Acute myocardial infarction (AMI) is uncommon in patients younger than 45 years of age. The cause for AMI in these patients can often be divided into two categories, angiographically-normal coronary arteries and those that are abnormal [1]. The etiology is often unclear in patients with no evidence of coronary artery abnormality, as seen in our patient. An AMI in a normal coronary artery with no evidence of atherosclerosis, spontaneous dissection, aneurysm, or other structural abnormalities can have various reasons. The pathophysiology of the AMI can sometimes be explained on the basis of coronary artery spasm, thrombosis, embolization, or a mixture of these processes [1-2]. Coronary vasospasm from recreational drugs such as cocaine and amphetamine has been well studied and documented. Cases of AMI from severe coronary vasospasm have been reported with phentermine, a weight loss medication and an amphetamine derivative, but coronary artery thrombosis has not [3-4]. However, the literature review does show the possibility of coronary artery thrombosis secondary to endothelial damage from prolonged severe coronary vasospasm in patients without evidence of atherosclerotic plaque rupture [5-11]. More recently, coronavirus disease 2019 (COVID-19) has been shown to be associated with various thromboembolic phenomena, however, the majority of cases involving acute coronary artery occlusion have been observed in hospitalized patients with concurrent respiratory failure [12-17]. Post-infection coronary artery thrombosis in a recovered asymptomatic patient has yet to be reported. In this case report, we present a 23-year-old female who presented with an acute anterior-lateral ST-elevation myocardial infarction (STEMI) and was found to have a laminated thrombus in her left anterior descending coronary artery (LAD). She tested negative for severe acute respiratory syndrome coronavirus 2 (SARS-CoV-2), however, she was found to be IgG positive on COVID-19 serology, indicating a remote history of asymptomatic infection. She also was taking phentermine for weight loss. Although the mechanism by which our patient developed this thrombus remains unclear, our report provides evidence for two possible culprits, COVID-19 and phentermine.

## Case presentation

Our patient is a 23-year-old African American female with a past medical history of obesity (body mass index of 33.8) who presented to the emergency room with progressively worsening substernal chest pain that started one hour prior to presentation. The chest pain radiated down both arms, was crushing in nature, and was associated with one episode of non-bloody emesis. She denied any prior cardiac history and recently arrived back from West Africa over a month ago. She denied any history of exertional or rest angina, dyspnea, orthopnea, lower extremity edema, palpitations, or abdominal pain. Family history was significant for diabetes mellitus in the biological father. There was no history of myocardial infarction, sudden cardiac death, premature coronary artery disease (CAD), cardiomyopathy, stroke, or a thromboembolic disorder in the patient or her family. She denied any recent illness or close contact with anyone who was sick or known to be positive for COVID-19. She had no history of symptoms associated with COVID-19 such as fever, dry cough, chills, myalgia, anorexia, diarrhea, and loss of taste or smell. She was never hospitalized prior to this admission. The patient reported taking phentermine 30 mg daily for weight loss for approximately one year and had a levonorgestrel intrauterine contraceptive (IUD) device placed two years ago. She denied any alcohol, tobacco, or illicit drug use.

**Figure 1:**
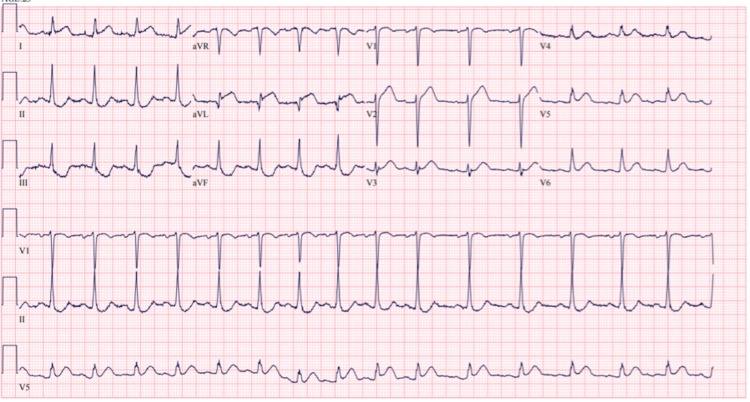
Initial electrocardiogram Initial ECG done at the time of arrival to emergency room showing sinus tachycardia and minimal ST elevation in leads I and avL with mild ST depression in leads II, III, aVF.

**Figure 2:**
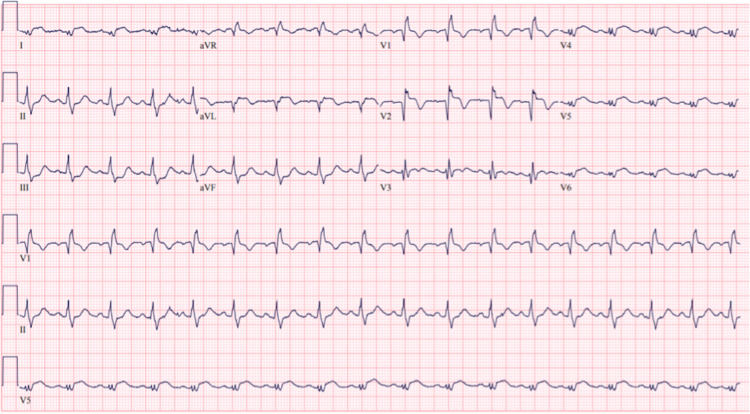
Subsequent electrocardiogram Repeat ECG done approximately 30 minutes after initial ECG showing prominent ST-segment elevation in leads I, avL, V2-V6, reciprocal ST segment depression in leads II, III, avF, and new T wave inversions in leads V1-V2.

**Figure 3:**
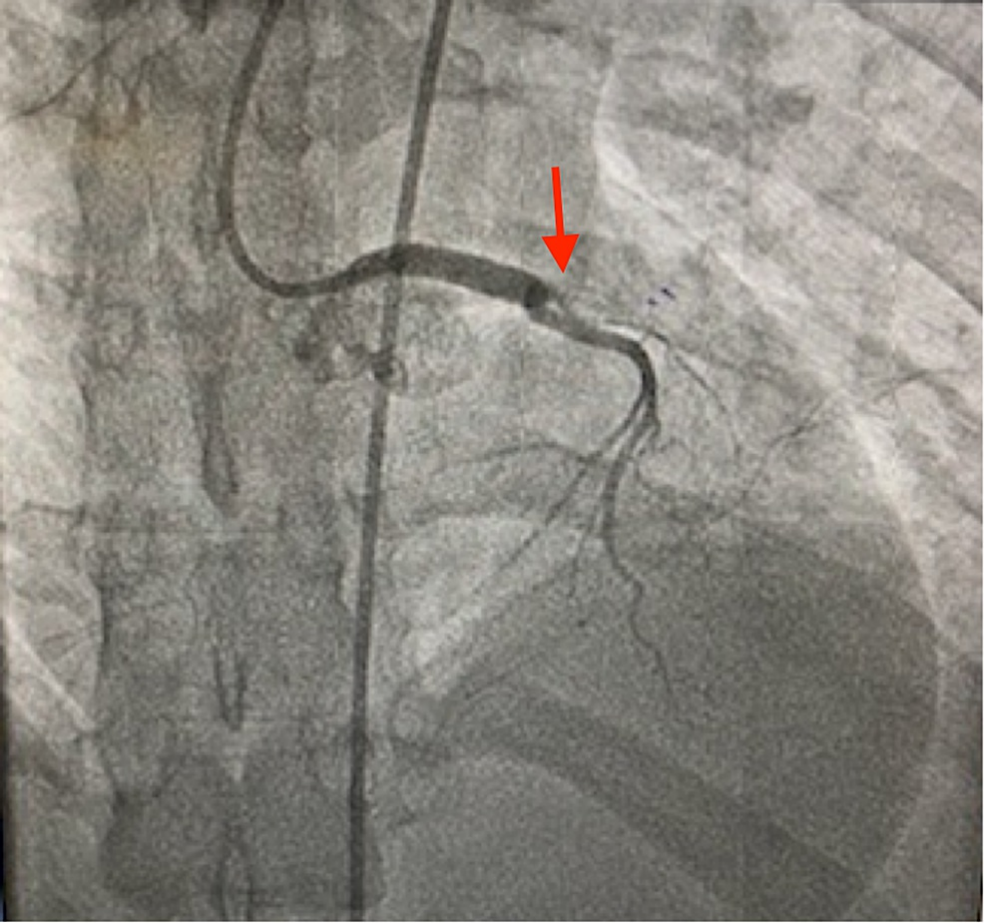
Left anterior descending coronary artery (LAD) occlusion Coronary angiography showing complete occlusion of LAD (red arrow).

**Figure 4:**
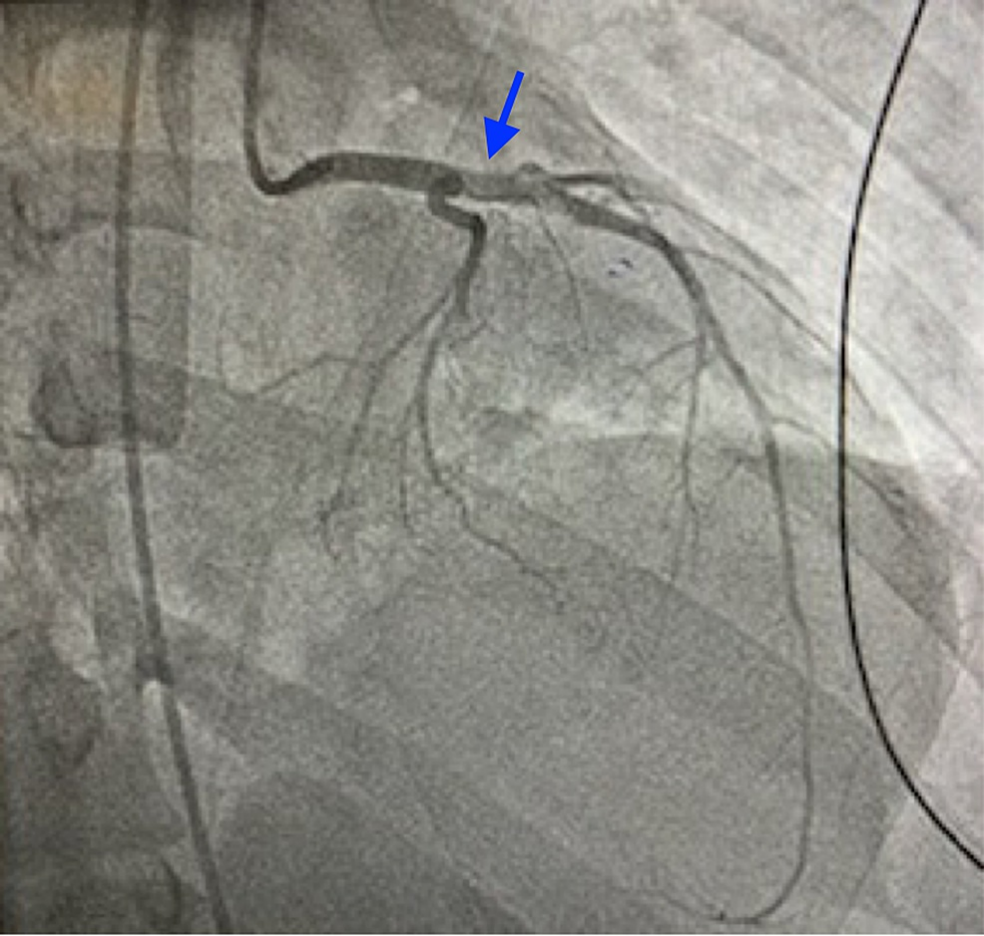
Post-intervention left anterior descending coronary artery (LAD) angiography After successful aspiration thrombectomy of the LAD thrombus, significant improvement in coronary blood flow was seen (blue arrow).

Her vitals on arrival were 107/76 blood pressure, heart rate 100, respiratory rate 14, oxygen saturation 97% on room air, and temperature 98.6. Physical exam showed normal and equal peripheral pulses in upper and lower extremities, no chest wall tenderness on palpation, normal S1 and S2 heart sounds, and clear lung fields. There was no evidence of accessory respiratory muscle use, lower extremity edema, jugular venous distention, or chest wall trauma. She received aspirin 325 mg, 4 mg of morphine, 4 mg ondansetron, and 40 mg pantoprazole in the emergency room. Initial work up revealed a normal troponin level and electrocardiogram (ECG) showing sinus tachycardia, minimal ST-segment elevation in lead I and aVL, and slight ST-segment depression in lead II, III, and avF (Figure 1). CT angiogram of the chest was negative for pulmonary embolism, aortic dissection or parenchymal opacity, or infiltrates. She had a rapid nasopharyngeal (NP) Xpert (Cepheid, Sunnyvale, CA) SARS-CoV-2 swab to screen for COVID-19, which was negative. Her chest pain continued to worsen during her initial work up thus repeat ECG was done which showed prominent ST-segment elevation in anterior leads with reciprocal depression in inferior leads, which were new compared to the previous ECG (Figure 2). Repeat troponin levels increased to 0.081 compared to baseline troponin of 0.012 (Table 1). The patient was taken to the catheterization laboratory for emergent coronary angiography for STEMI. Access was obtained through the right common femoral artery and with the first injection, total occlusion of the LAD was revealed (Figure 3). Balloon angioplasty of the proximal LAD was attempted, however, no improvement in flow was seen thus aspiration thrombectomy was attempted however no reflow was observed, despite multiple runs. Intra-coronary adenosine and nicardipine were given, which resulted in some improvement in flow (Figure 4). Repeat aspiration thrombectomy was performed with removal of large clots resulting in significant improvement in flow in the LAD. Intravascular ultrasound (IVUS) of the proximal LAD did not show any evidence of atherosclerosis or plaque, stenosis, or dissection. Immediately post-cardiac catheterization, the patient went into acute cardiogenic shock with flash pulmonary edema requiring rapid sequence intubation, vasopressor support, and insertion of an intra-aortic balloon. Patient was transferred to ICU where she developed acute kidney injury and shock liver secondary to hypoperfusion from cardiogenic shock requiring epinephrine, norepinephrine, vasopressin, and dobutamine drips. Transthoracic echocardiography (TTE) showed severe left ventricular hypokinesis with an estimated left ventricular ejection fraction (LVEF) of 10%. Patient was transferred to a tertiary care center for immediate venoarterial extracorporeal membrane oxygenation (VA ECMO). She was able to be weaned off of all vasopressors after initiation of VA ECMO. Approximately 1-2 weeks after transfer, she was able to be taken off VA ECMO and was extubated. Her repeat TTE on discharge showed improvement in LVEF to 30%-35%.

**Table 1:** Search for thrombus etiology: medical work up Laboratory analysis was aimed at stratifying cardiovascular risk factors and for ruling out common hypercoagulable disorders, rheumatological diseases, and vasculitides.

Complete blood count (CBC) with differential	Unremarkable
Comprehensive metabolic panel (CMP)	Unremarkable
Troponin Levels (ng/mL)	On arrival: 0.012, thirty minutes after arrival: 0.081, post PCI: 21.450, prior to transfer to tertiary center: 13.930
Lipid panel (mg/dL)	Cholesterol: 132, triglycerides: 132, high-density lipoprotein (HDL): 59, low-density lipoprotein (LDL): 61
Hemoglobin A1C (HbA1c)	5.60
Thyroid stimulating hormone (mU/L)	3.23
D-Dimer (mcg/mL)	2.33
C-Reactive protein (mg/dL)	4
Erythrocyte sedimentation rate (mm/hr)	12
Ferritin (ng/mL)	122
Pro-thrombin time (sec)	13.6
Activated partial thromboplastin time (sec)	23.6
International normalized ratio (INR)	1
Fibrinogen (mg/dL)	475
Homocysteine (Umol/L)	6.9
Factor V Leiden	Negative
Antithrombin III (percentage)	80
Activated protein C resistance	2.4
Protein C (percentage)	105
Protein S (percentage)	46
Factor V activity (percentage)	56
Factor VIII activity (percentage)	78
Anti-Factor Xa (IU/mL)	0.68
Complement (C3/C4) level (mg/dL)	92/30
Total complement CH50	Normal
ANA titer	<1:80
ANCA screen with reflex to titer	Negative
Lupus anticoagulant (IgM/IgG)	Negative
B2 Glycoprotein (IgM/IgG/IgA)	Negative
Anti-cardiolipin (IgM/IgG/IgA)	Negative
Rheumatoid factor U/mL	25

Cardiovascular risk factors such as hyperlipidemia, diabetes, and thyroid dysfunction were ruled out with laboratory testing (Table 1). Urine and serum beta human chorionic gonadotrophin (hCG) were negative. Urine toxicology was negative for cocaine, marijuana, opiates, phencyclidine (PCP), and amphetamines. Infectious work up with urine analysis with microscopy, blood cultures, respiratory viral panel, and malaria/parasitic blood smear were all negative. Testing for sexually transmitted infections such as human immunodeficiency virus (HIV), hepatitis, syphilis, herpes, gonorrhea, chlamydia, and trichomonas was also negative. Hypercoagulable conditions such as nephrotic syndrome, antiphospholipid syndrome, systemic lupus erythematosus (SLE), factor V leiden were tested for and ruled out. Conditions that can cause accelerated atherosclerosis such as familial hypercholesterolemia, hypertriglyceridemia, and hyperhomocysteinemia was also ruled out. Other rheumatological disorders, as well as vasculitis, were also ruled out (Table 1). Transesophageal echocardiography (TEE) did not show any valvular vegetation or intracardiac shunt to suggest a possible embolic phenomenon. Her chest imaging was negative for any ground-glass opacities typically seen in COVID-19 pneumonia. Inflammatory markers such as D-dimer, C-reactive protein (CRP), erythrocyte sedimentation rate (ESR), and ferritin, typically elevated in COVID-19 infection, were all normal on admission. During the course of her hospital stay, she was screened a total of three times for COVID-19 using the NP SARS-CoV-2 swab and once with bronchoalveolar lavage (BAL) SARS-CoV-2 sampling, all of which were negative. Samples collected via BAL and NP were tested using real-time polymerase chain reaction (PCR).

The patient was ultimately discharged home; one-month post-discharge follow up revealed she tested positive for SARS COV-2 IgG but negative for IgM. Vaccination against SARS COV-2 was not available to the public nor was it approved by the FDA at the time the patient was tested for SARS COV-2 antibodies.

## Discussion

Given the rare incidence of STEMI in a young female in her 20s with no structural coronary artery abnormalities such as aneurysms, ectasia, or dissection on coronary angiography, and no evidence of atherosclerosis or plaque on intracoronary ultrasound, a hypercoagulable and rheumatological work up was done to investigate the cause of coronary artery thrombus formation (Table 1). Work up did not reveal any autoimmune, infectious, or hypercoagulable disorders. Cardiovascular risk factors for AMI in a young patient are male gender, smoking, recreational drug use, hypertension, low high-density lipoprotein (HDL), obesity, and high triglycerides [1-2]. Cardiovascular risk factors for our patient included obesity and phentermine use for weight loss. Another possible risk would be oral contraceptives, however, our patient had a levonorgestrel IUD, which has been shown to not have an increased risk for venous or arterial thrombosis [18-19]. Another uncommon but frequently seen condition in young females presenting with STEMI is spontaneous coronary artery dissection (SCAD) [20], however, our patient did not have evidence of dissection on coronary artery angiography or IVUS.

Phentermine, like other amphetamines, has the potential to causing myocardial ischemia, most commonly from coronary vasospasm [3]. To date, there have not been any reports of phentermine or other amphetamines having pro-thrombotic activity. However, severe coronary vasospasm has been reported [3-4]. There have been few reports of severe coronary vasospasm-induced thrombus formation secondary to possible endothelial damage [5-10]. Endothelium of the coronary arteries inhibits platelet aggregation by formation and release of prostacyclin, and it reacts to platelet products by causing relaxation of the underlying smooth muscle. In addition, if any thrombin is formed, it also causes endothelium-mediated relaxation. If the endothelium is damaged, these protective mechanisms are lost. Patients with coronary artery spasm usually have morphologic changes in the artery at the site of the spasm. Platelets can aggregate at this site and release vasoactive substances, which-aided by formation of thrombin-cause contraction [6,8-10]. Literature shows coronary vasospasm occurring usually within one month of phentermine therapy [3-4]. Given our patient's one-year history of phentermine use, thrombus formation from endothelial damage secondary to severe vasospasm may be less likely.

Our patient was admitted during the early stages of the COVID-19 pandemic outbreak and, given its association with thromboembolism and hypercoagulability, the patient remained under isolation during the course of her hospitalization. She did not have any signs or symptoms or COVID-19 except for a recent travel history to West Africa. Typical COVID-19 signs and symptoms, ground-glass opacity on CT imaging, and elevated inflammatory markers were absent in our patient. She tested negative multiple times and given the unavailability of the COVID-19 serology testing at the hospital, the test was not obtained during her hospital stay. The patient obtained her COVID serology one month post discharge and tested positive for SARS COV-2 IgG. Given her positive serology, it is reasonable to assume that at some point before her STEMI, she was infected with COVID-19; however, she was completely asymptomatic. Cardiac injury from COVID-19 is not well understood, however, the majority of cases reported involve patients with a severe form of the disease, usually requiring hospitalization [11-17]. COVID-19 is associated with hypercoagulability both during acute infection and after recovery 14 [11]. Recent reports have shown arterial thrombosis in the heart, brain, and limbs in recovered patients that experienced only mild disease [15-17], however, coronary artery thrombosis in an asymptomatic COVID-19 carrier has never been reported. We believe the likely etiology of our patient's LAD thrombus is COVID-19.

## Conclusions

Our patient suffered from a near-fatal STEMI at a young age with no underlying cardiovascular risk factors except for obesity. A laminated thrombus was aspirated from an anatomically normal LAD. Extensive work up for hypercoagulable conditions was unremarkable. Phentermine use remains a suspect as well-established data shows induction of coronary vasospasm, however, given our patient’s chronic use of this medication, the diagnosis cannot confidently be made. Although coronary artery thrombosis post COVID-19 is asymptomatic, carriers have not been reported; it is our belief that our patient’s STEMI will be the first case to show this possible association.
